# Newborn Hearing Screening: Analysing the Effectiveness of Early Detection of Neonatal Hearing Loss in a Hospital in Greece

**DOI:** 10.7759/cureus.19807

**Published:** 2021-11-22

**Authors:** Panagiota Kosmidou, Sotiris Tzifas, Spyros Lygeros, Gerasimos Danielides, Thomas Nikolopoulos, Gabriel Dimitriou, Stavros Angelis, Stefanos Naxakis

**Affiliations:** 1 Department of Otorhinolaryngology-Head and Neck Surgery, University of Patras School of Medicine, Patras, GRC; 2 Department of Pediatrics and Neonatology, University of Patras School of Medicine, Patras, GRC; 3 Second Ear-Nose-Throat Department, Attikon University Hospital, National and Kapodistrian University of Athens, Athens, GRC; 4 Department of Surgical Anatomy, National and Kapodistrian University of Athens Medical School, Athens, GRC

**Keywords:** newborns, transient evoked otoacoustic emissions, efficacy, hearing loss, newborn hearing screening

## Abstract

Introduction

The introduction of newborn hearing screening programs (NHSP) has drastically contributed to the early diagnosis of hearing loss (HL) in children, with the prospect of children developing speech as early as possible. This retrospective study aims to present and discuss the preliminary results of the NHSP at the University Hospital of Patras, Greece, highlighting the strengths and weaknesses of the program. The evaluation of the implementation of NHSP is important to confirm the effectiveness of the process and elaborate system failures.

Materials

The study describes the results of previous data collected from the NHSP in the Rio hospital of Patra and analyzed the conditions of the sample collected. The random sample involved newborns born between November 2018 - December 2020 at the University Hospital in Patra, Greece, which was assessed using transient evoked otoacoustic emissions (TEOAEs). Testing was performed twice per week on Thursday and Friday with a random sample, specifically examining the babies in the hospital these days. From the 2014 newborns assessed, 1491 were healthy neonates, while the other 523 required hospitalization in the neonatal unit.

Results

In total, there were 2014 live births; 1491 healthy neonates were screened with TEOAEs. Of them, 44 did not pass the first test. After retesting one month later, 31 passed the test, while the other 13 were referred to a hearing centre for further audiological testing with auditory brainstem response (ABR) tests. Two infants never showed up for the follow-up appointment. Of the remaining 11, six infants had normal hearing, three had otitis media with effusion or other conductive HL. The last two infants had HL. Specifically, one had bilateral sensorineural HL greater than 40db, and one had unilateral sensorineural HL greater than 40db.

Risk factors were identified in 523 newborns admitted to the unit. The most common risk factors identified were the use of ototoxic drugs, low Apgar scores, and prematurity. Of all the newborns, 491 passed the test the first time, and the rest 32 infants came back 1-2 months after leaving the neonatal unit. All the babies who had failed in the first screening test appeared for the follow-up appointment for the second screening test. Of these, 24 babies passed the test, but eight did not. Of these, four were diagnosed with media otitis with effusion or other conductive HL. Sensorineural HL was identified in the last four babies using ABR tests. In detail, two had unilateral sensorineural HL greater than 40db, while two had bilateral sensorineural HL greater than 40db.

Conclusion

In conclusion, we found that for the NHS programs to be effective, they must be implemented long-term and have monetary support. Early diagnosis and cochlear implantation are the keys to excellent outcomes. Cooperation between different specialties and a patient-centred approach will help physicians holistically face neonatal HL. Building trust between the parents and doctor is essential for the program's success and reducing the lost-to-follow-up rate. To run a successful program, trained staff, equipment, and financial support are required. However, the gold standards for the success of the program are proper implementation of the program, close follow-up, strict adherence to the guidelines in the neonatal intensive care unit (NICU), and the early detection and diagnosis of HL.

## Introduction

The recent implementation of neonatal hearing screening (NHS) programs in many countries has allowed for the early detection of congenital hearing loss (HL). HL is the most common genetic disorder in England and the United States, with a prevalence of 1.33 per 1,000 neonates and 1.86 per 1000 neonates at birth respectively [1-4]. The prevalence increases with age. During childhood, it is 2.7 per 1000 children which increases to 3.5 per 1000 in adolescence [1]. Hearing is an integral part of language development, and HL negatively impacts language skills and children's behavioural and emotional development [5]. Early detection encourages the treatment of neonates with hearing aids or cochlear implants as early as possible [5]. Current guidelines suggest initiating treatment no later than 3-6 months. In the future, there are plans to reduce the timeline to a confirmed diagnosis by two months of age and treatment no later than three months [6]. Early diagnosis of infants with HL is vital and is associated with more success in school and better social and cognitive development [7].

In Greece, the implementation of the program began in 2009, with many hospitals in the public and private sector adopting the screening program. This study aims to analyze the effectiveness of the neonatal hearing screening program in a hospital in Greece and the factors that contribute to the success of the program.

## Materials and methods

The Institutional Review Board (IRB) of the University of Patras Medical School, Patras, Greece issued approval 722/19-11-2018 for this retrospective study.

The method used for data collection was a questionnaire completed by the parents. To carry out the present research, the quantitative method was applied. In detail, the questionnaire inquired about the factors that may have affected the level of hearing of newborns and the methodology followed was the "research process". The results were quantified on a Likert scale in which 1 corresponds to a positive answer (Yes) while 2 to a negative one (No).

For the documentation of the theoretical framework, a search was performed in international Greek and English bibliographies in databases such as "Google Scholar".

The data collection was conducted electronically, and the parents entered their phone numbers voluntarily. Parental consent was gained, and data confidentiality and protection were maintained throughout the study.

To perform the transient evoked otoacoustic emissions (TEOAEs) assessments, the MADSEN Accuscreen handheld Otoacoustic Emissions Screener (GN Otometrics, Taastrup, Denmark) was used [8]. The Accuscreen was connected to a personal computer, and the AccuLink software program (GN Otometrics, Taastrup, Denmark) recorded the TEOAEs. The test took less than 10 minutes and was performed in quiet conditions by the doctor and nurse. Before beginning the assessment, the instrument calibrates automatically, and the test starts when calibration finishes. Upon completion of the examination, the Accuscreen displays a Pass/Refer response. The pass response is acceptable when [8]:

● The test registers more than eight valid peaks in opposite directions.

● The artefact value is less than 20%

● The quality (stability) of the probe position is more than 80%

When the test does not meet the above criteria, it is considered a 'Failed' test, and the word 'Refer' is displayed on the monitor.

The Universal Neonatal Hearing Screening guidelines are as below. Initially, babies undergo TEOAEs test and if they fail the first test within the first month, the test is repeated. Failing the second test, at an age of 4-6 weeks an automated auditory brainstem response (AABR) test is performed. At 3-6 months, hearing aids for mild or moderate HL and sign language education for the family are recommended. Finally, at 10-12 months cochlear implants for severe HL are recommended [9].

In our study, 2014 neonates were evaluated using TEOAEs as a screening test for HL. TEOAEs measure the total outer hair cell response from auditory inputs in the cochlea [10]. The TEOAEs test is a highly sensitive (80% - 96.5%), and specific test (90.60% - 92.85%) [11,12].

All neonates in the study were born in the University Hospital of Patras, Greece, from November 2018 to December 2020. The sample was random and involved newborns tested in the hospital's maternity clinic, with the department's permission, only some specific days per week (Thursday and Friday).

**Figure 1:**
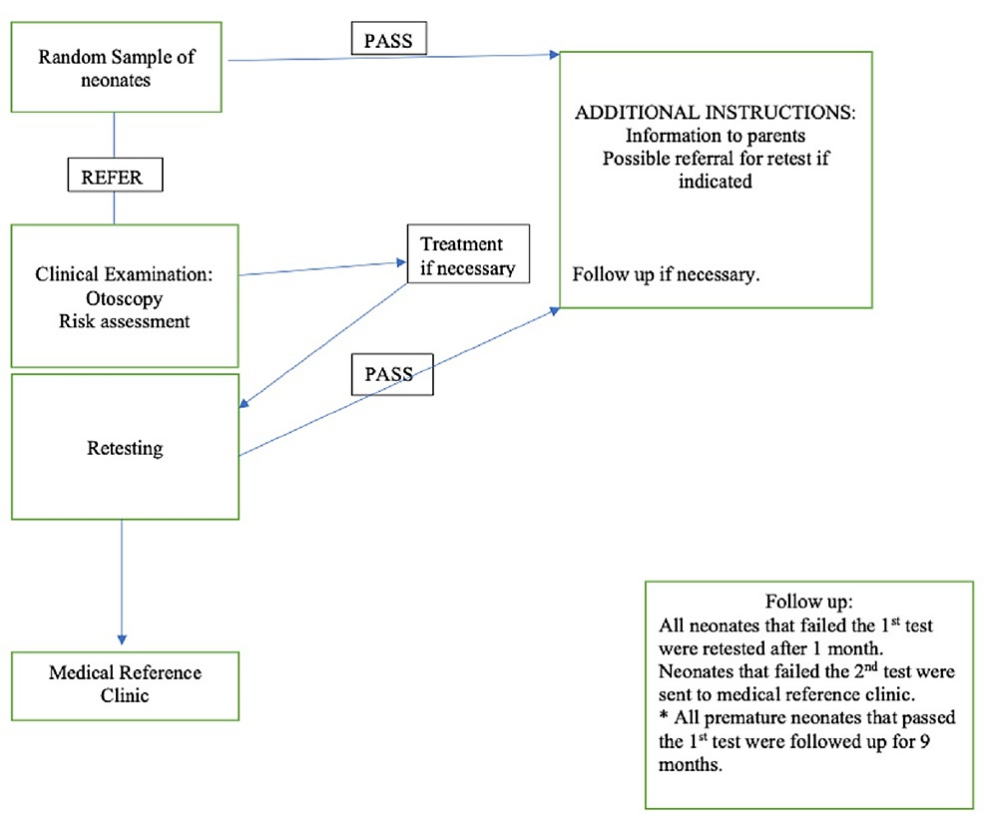
Pathway design for TEOAEs test of neonates. TEOAE; transient evoked otoacoustic emission

The sample was comprised of 1491 healthy neonates and 523 neonates which were admitted to the neonatal intensive care unit (NICU). The first TEOAEs test was performed during the first days of life. However, the neonates that failed the first test were retested within the first month. An important distinction is that premature neonates were followed until nine months of age (Figure 1). Failure of both the first and second TEOAEs tests led to a referral and further investigations at audiology centres and underwent auditory brainstem response (ABR), radiology (CT and MRI), infection (Toxoplasma gondii, rubella, cytomegalovirus, herpes simplex virus, syphilis), genetic (Connexine 26), vision (fundus, electroretinography), kidney, heart, and thyroid function testing [6].

## Results

**Figure 2:**
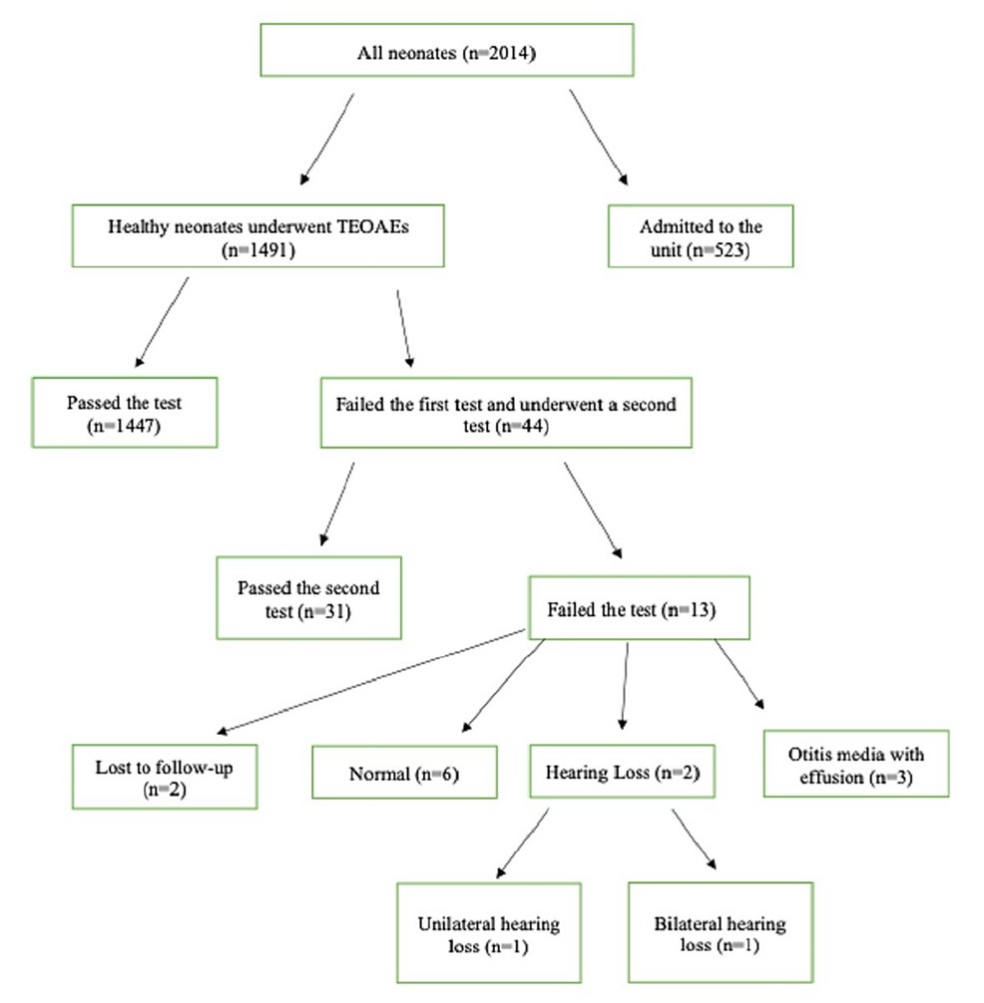
Illustration of the screening process of healthy neonates. TEOAE; transient evoked otoacoustic emission

Overall, 1491 healthy neonates were screened, of which 44 failed the first TEOAEs screening test. The neonates that failed the initial test were re-examined within the first month. Upon re-examination of the babies, 31 were found to be normal and 13 again failed TEOAEs and were subsequently referred to the Medical Reference Clinic for further audiologic investigation. Unfortunately, the parents of two neonates that did not pass the second test never communicated and did not show up for the follow-up appointments. Of the remaining 11 children that failed both tests, six had normal hearing, and three had otitis media with effusions or other conductive HL. The last two newborns were diagnosed with HL using ABRs. More specifically, one had bilateral sensorineural HL greater than 40db, and one had unilateral sensorineural HL greater than 40db (Figure 2).

**Figure 3:**
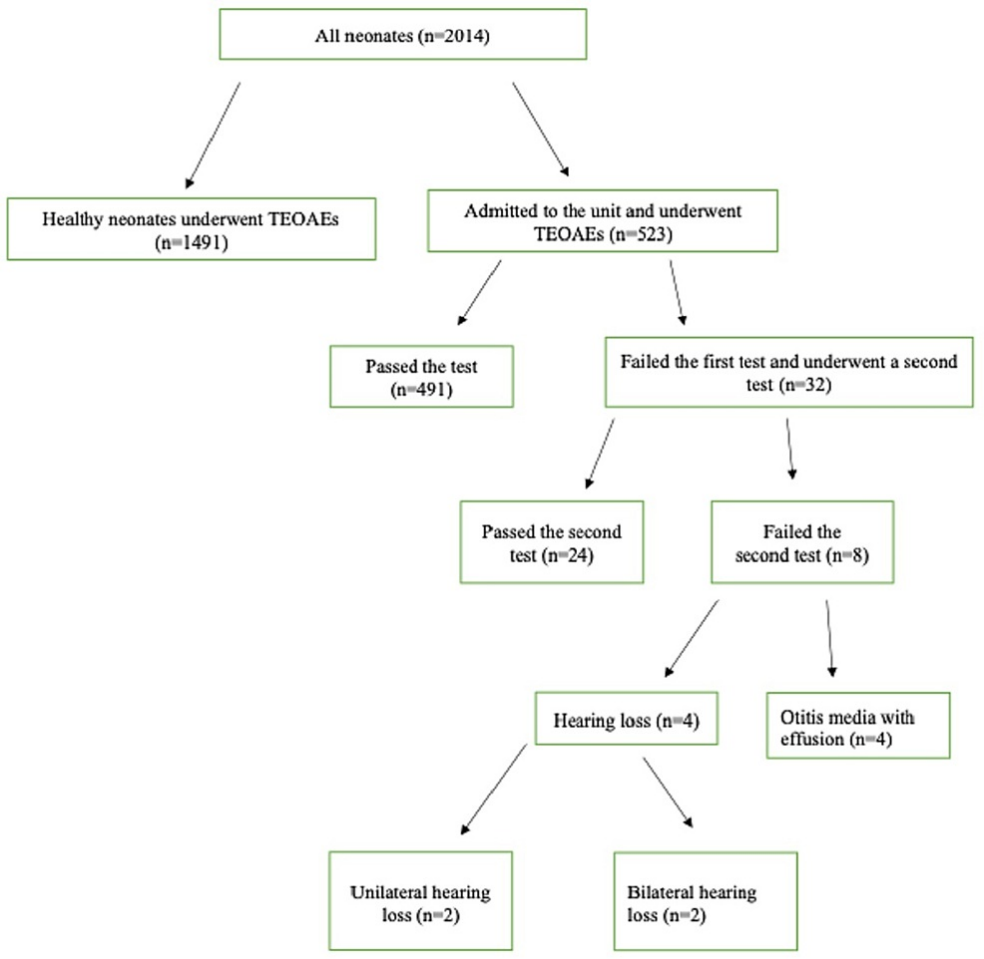
Illustration of the screening process of neonates admitted to the unit. TEOAE; transient evoked otoacoustic emission

The research assessed the 523 premature newborns that were admitted to the NICU. The most prominent risk factors of neonatal HL described in current guidelines are the use of ototoxic drugs for more than five days, prematurity, mechanical ventilation for more than five days, hyperbilirubinemia with exchange transfusion regardless of the length of stay, and low Apgar scores which assess for signs of hemodynamic compromise [6,13-16]. After the first TEOAEs were performed, 491 of the newborns passed the test, and 32 did not. The 32 children who had failed in the first screening test appeared for the follow-up appointment for the second screening test. There were no babies lost-to-follow-up. Additionally, 24 babies of these passed the test, but eight did not. Of these eight babies, four failed the second test and were diagnosed with otitis media with effusion or other conductive HL. The last four newborns were diagnosed with sensorineural HL after ABRs tests. In detail, two had unilateral sensorineural HL greater than 40db while two had bilateral sensorineural HL greater than 40db (Figure 3).

**Figure 4:**
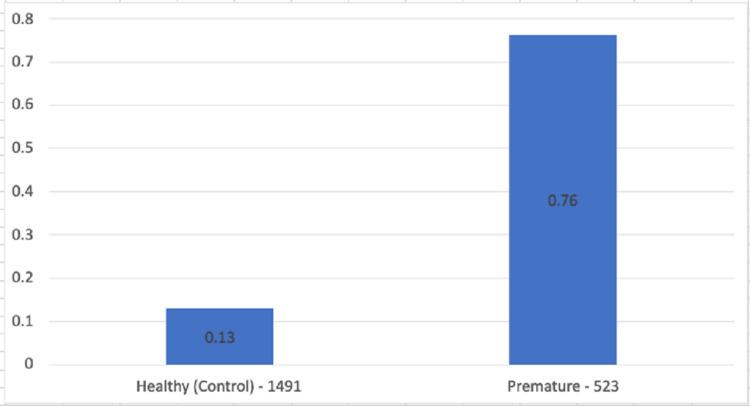
Hearing loss percentage.

In our study, the percentage of HL in healthy neonates was 0.13%, and for premature neonates 0.76% (Table 1) (Figure 4).

**Table 1:** Hearing loss percentage.

	Neonates	Hearing loss	Total Percentage
Healthy (Control)	1491	2	0.13
Premature	523	4	0.76

## Discussion

The introduction of screening programs worldwide aimed at early HL detection to promote normal child development. However, several issues have arisen.

A significant disadvantage of neonatal hearing screening programs is the high false-positive rate [17]. It is concerning that the false-positive rate ranges from 0.3% to 16.7%, with a false diagnosis creating anxiety to many parents [18,19]. According to Clemens et al., adding a second test before the discharge of infants that had failed the initial screening, reduced the false-positive rate from 3.3% to 1.9% [20]. Other literature states that performing a second TEOAEs test before discharge or after discharge, but before the subsequent audiological examination, significantly reduces the false-positive rate [20,21].

The second major weakness of the NHS programs is the large number of newborns lost to follow-up [17]. In our study, the lost-to-follow-up rate was 0.09%. Aidan et al. found that the babies' lost-to-follow-up percentage in a hospital in Paris, France is 48.3%, while a large German study by Rohlfs et al. found the percentage to be 31.3% [18,22]. A significant factor for the sizeable lost-to-follow-up percentage is parental attitude [23]. The parents' reluctance and fear of potentially severe disease, stigma, or other psycho-social reasons lead to missed consultations, and as a result, many neonates do not receive treatment [23]. To overcome this phenomenon, physicians should educate parents in a friendly manner and help them understand the importance of follow-up appointments in identifying and preventing HL. Implementing measures such as a tracking system with ID numbers, names, and telephone calls, as well as dedicated secretariat support, have the potential to reduce the lost-to-follow-up rates [7]. The screening program and protocols must respect the private information of all patients according to doctor-patient confidentiality.

Additionally, for the screening program to be effective, during our study we found that cooperation and effective communication between otorhinolaryngologists and the paediatricians, the nursing staff, and audiology technicians are essential. If HL was diagnosed, psychologists and speech therapists were included in the diagnostic team. A difficulty faced in our study was coordinating the personnel of different occupations and ensuring the patient's best care. Also, trust between parents and the hospital is of the highest importance, especially for the follow-up of the neonates, to minimize the danger of undiagnosed HL. An interesting fact was that during this study, it was noted that the parents whose children were previously admitted to the NICU, were more likely to show up at follow-up appointments.

In the NICU, after the neonate’s discharge, the hospital's protocol instructions for the parents were: regular pediatric visits including vaccinations, breastfeeding or, if not possible, formula milk, administration of Vitamin D, Iron, and Folic acid as needed for healthy neonates [24]. All newborns are tested using TEOAEs and ABRs before their discharge. Neonates admitted to the unit, along with the above instructions, were suggested to undergo an HL test if one was not performed prior, a brain ultrasound, a fundoscopic examination, and to be closely followed by a neurodevelopmental paediatrician until the age of 4 to 5 years.

It is important to note that no syndromic cases of HL were included in our study due to the extensive genetic testing performed in the hospital. The detailed prenatal genetic testing complements the neonatal HL program, as it reduces total HL diagnoses by minimizing the incidence of syndromic cases of HL.

An essential constituent for the success of the program after discharge is the role of the paediatrician. The paediatrician is responsible for observing the children's neurodevelopmental milestones, including hearing, for the first few years of life. We found that close collaboration between the otorhinolaryngologist and paediatrician led to more HL diagnoses, and parents were more eager to attend the follow-up appointments. It is crucial to treat HL, either with hearing aids or cochlear implants, depending on the nature and severity of the HL [25]. However, the program does not stop at treating audiological issues in children. The program's reach should be to help children fit into society and have an everyday life. To achieve this, money, time, and resources must be allocated to help these children.

Analyses regarding the cost of the NHS vary; however, the price is an essential factor regarding the permanent implementation of the program. An extensive and detailed study by Ciorba et al. estimated the cost at €13.86 per infant in Italy [26]. The above value differs according to the country, as well as the protocol and the equipment used. The main factor of variation in cost in programs worldwide is the salary of the persons involved in the program, such as doctors, nurses, and technicians [26]. There is a lack of literature analyzing the cost of NHS programs, especially in Europe, and more studies are needed to gain a more accurate estimate of the actual cost. The most frequent problem of our program is severe understaffing, which affects the permanent implementation of the program. More doctors, nurses, and audiology technicians were required for the program to be run effectively. In the current climate, due to economic restrictions and the nationwide economic crisis, early retirement, and no new hiring, the program struggled to be implemented without increasing the quantity and quality of staff. Thus, as is true for many countries worldwide, financial support is essential to materialize the program.

The preferred treatment for HL is hearing aids or cochlear implants; both are beneficial [27]. Early diagnosis and cochlear implantation are key for excellent outcomes. Currently, the suitable age for cochlear implantation is >12 months of age but there are plans to reduce this and allow for cochlear implantation <12 months to achieve a standard of care status, highlighting the importance of early intervention [6].

Furthermore, as mentioned above, although prematurity is not mentioned in the Joint Committee on Infant Hearing (JCIH), it has been mentioned as a prominent risk factor for HL by several investigators [6,28,29]. According to current literature, neonatal HL is estimated at 2%-3% of premature neonates and 0.1%-0.2% in healthy neonates [30]. In our study, the percentage of HL in healthy neonates was 0.13%, and for premature neonates, it was 0.76%. Thus, our study corroborates that prematurity is a considerable risk factor regarding neonatal HL. However, it is important to note that we had a smaller sample than other studies, so it is difficult to generalize to further studies. Moreover, our study assessed one hospital in Greece, and we cannot make assumptions about hospitals worldwide.

Finally, our study begs the question as to whether it is more efficient and cost-effective to screen only premature infants for cost purposes and the very low prevalence of neonatal HL in full-term infants present in the current literature, but there is the risk that healthy neonates remain undiagnosed [30].

A limitation of our study was the small sample. Although the study successfully detected HL in our hospital, a more significant number of neonates tested is required to generalize our results. Also, the specific guideline protocols depend on the healthcare system of each country. For example, the initial screening test used is only ABR or TEOAEs, and others use both. Thus, it is difficult to compare with other countries, as the protocol is different.

## Conclusions

In conclusion, we found that for the neonatal hearing screening programs to be effective, they must be implemented long-term and have monetary support. Early diagnosis and cochlear implantation are key for excellent outcomes. Cooperation between different specialties and a patient-centred approach will help physicians holistically face neonatal HL. Building trust between the parents and doctor is essential for the program's success and decreasing the lost-to-follow-up rate. To achieve a successful program, we must have more trained staff, equipment, and financial support. Finally, the gold standard for the success of the program is proper implementation, close follow-up, strict adherence to the guidelines in the NICU, and early detection and diagnosis of HL.
